# Supplementary figures and images for: Characterizing rurality using the All of Us Research Program data
Source: PLoS One. 2025 Dec 4;20(12):e0328958. doi: 10.1371/journal.pone.0328958 (PMC12677462; doi:10.1371/journal.pone.0328958)

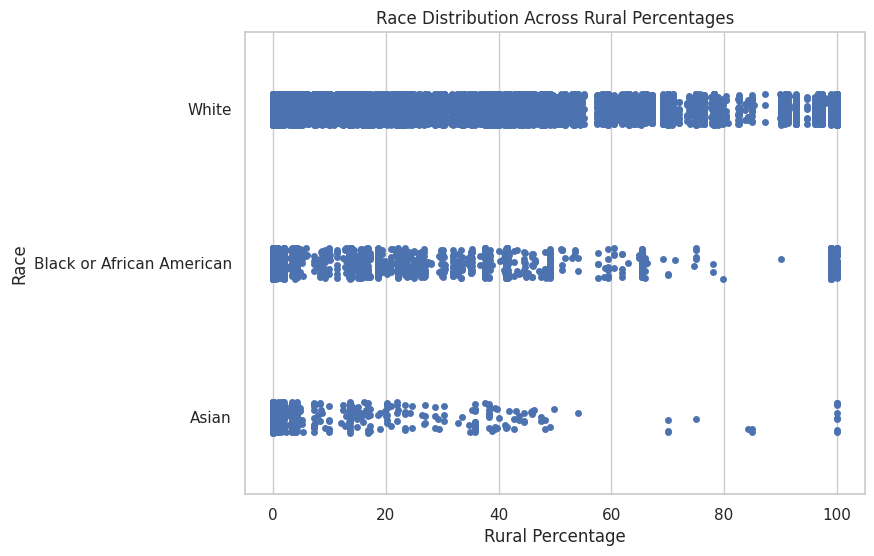

Supplement: S1 Fig — There is a decrease in racial diversity with increasing rural percentages. (TIF) [file pone.0328958.s001.tif]
