# Supplementary material for: Characterizing rurality using the All of Us Research Program data
Source: PLoS One. 2025 Dec 4;20(12):e0328958. doi: 10.1371/journal.pone.0328958 (PMC12677462; doi:10.1371/journal.pone.0328958)
Supplement: Attachment — Submitted filename: Comments - All of Us research program.docx [file pone.0328958.s002.docx]

**Comments**

**Introduction section**

The introduction moves logically from the general problem of rural health to the specific challenge of defining rurality, to the limitations of the *All of Us* dataset, and finally, to the study's proposed solution. However, while the paper clearly states the purpose, the final sentences of the introduction could be strengthened to more explicitly highlight the **broader implications** of the work. For example, in addition to informing future research, how might this rurality scale be used by policymakers or public health organizations? Also, the introduction uses several citations to support its claims. You could consider starting the introduction with a single, compelling statistic about the life expectancy gap between rural and urban populations to immediately grab the reader's attention and underscore the urgency of the problem.

**Methods**

This is a well-structured and detailed "Methods" section that clearly outlines the study's approach. It effectively explains the different tiers of the *All of Us* dataset and, most importantly, provides a transparent, step-by-step process for creating the rurality scale. To further strengthen the methods section, there should be a justification for the threshold. **For instance, i**n the section on "Healthcare access and utilization survey data preparation and analysis," the authors state that thresholds of "six or more affirmative responses" for delayed care and "nine or more affirmative responses" for affordability were selected to reflect a "high burden." This is a critical point that needs more robust justification. Authors should explain the rationale behind these specific numbers. Were they based on a pre-existing clinical standard, a statistical analysis (e.g., a percentile cutoff), or a consensus among the research team? Providing a clearer justification will strengthen the validity of the findings.

**There must be consistency in Language:** The term "rurality" is used, but the scale is also referred to as a "rural-urban continuum" in the figure caption. While these are related, using consistent terminology throughout the paper will prevent any potential confusion.

### Discussion Section

The discussion correctly re-establishes the context by referencing the existing literature on rurality definitions. It effectively highlights the study's unique methodological strength—the creation of a continuous rurality scale compatible with de-identified data. However, here is a suggestion for revision:

Consider reorganizing the discussion to maximize its impact. A potential flow could be:

1. **Summary of Key Findings:** Start with a concise summary of your most significant findings from the results section. For example, "This study shows that participants living in areas with higher rurality percentages, as defined by our novel scale, report statistically significant disparities in delayed care and healthcare affordability compared to their urban counterparts."
2. **Connection to Literature and Contribution:** Next, explain how these findings align with or contradict existing research. Reiterate your study's unique contribution—the development of a practical rurality scale—in this context.
3. **Limitations and Implications:** Acknowledge any limitations of your methodology (e.g., reliance on 3-digit ZIP codes). Then, discuss the broader implications of your findings for patient care, policy, and research.
4. **Future Work:** Conclude by providing a clear and detailed roadmap for future research using your developed scale.

**Conclusion**

This is a strong conclusion that effectively summarizes the study's key contributions. It brings the paper full circle by re-emphasizing the problem and presenting the proposed rurality scale as a viable solution.

**Critique of the Conclusions Section**

- **Strengths:** The conclusion is concise and effectively restates the study's main purpose and findings. It correctly identifies the potential of the rurality scale to expand the scope and quality of research within the *All of Us* program.
- **Areas for Improvement:**
  - **Specificity:** The conclusion mentions "potential and current gaps" and "associations between rurality, delayed care, and healthcare affordability" without directly stating the findings. It would be stronger to explicitly state what was found.
  - **Proposed Revision:** Start with a more direct summary of the results. "In this study, we developed a standardized approach to identify and characterize rural participants within the *All of Us* program and found that higher rurality was significantly associated with increased reports of delayed care and difficulty with healthcare affordability."
  - **"So What?" Moment:** The last sentence is good, but it can be enhanced to leave a lasting impression. What is the ultimate goal of this work? It's not just about "quantifying representation"—it's about improving health equity.
  - **Proposed Revision:** The final sentence could be more ambitious. This work provides a critical foundation for future investigations, not only into how rurality intersects with social determinants of health but also for developing targeted interventions to improve health outcomes and reduce health disparities in rural America.
