# Supplementary material for: Characterizing rurality using the All of Us Research Program data
Source: PLoS One. 2025 Dec 4;20(12):e0328958. doi: 10.1371/journal.pone.0328958 (PMC12677462; doi:10.1371/journal.pone.0328958)
Supplement: Attachment — Submitted filename: Response to Reviewers.docx [file pone.0328958.s003.docx]

**Response to Reviewers: Rebuttal Letter**

Thank you for giving us the opportunity to revise our manuscript entitled “**Characterizing rurality using the All of Us research program data” (Manuscript ID: PONE-D-25-36901).** We sincerely appreciate the time and effort the reviewers and editors have invested in providing constructive feedback, which has helped us improve the quality and clarity of our work. We have carefully considered each comment and revised the manuscript accordingly. Below, we provide a detailed, point-by-point response to all reviewers’ comments. For clarity, we have reproduced each comment below and followed it with our response. All changes are highlighted in the “Revised Manuscript with Track Changes” document.

**Response to Reviewer 1**

**Comment 1:** Thank you for the opportunity to review this important research. This is a well-prepared manuscript that addresses an important methodological issue in rural health research: the lack of consistent and privacy-compliant measures of rurality in large-scale datasets. The study’s strengths include its use of a very large, diverse cohort (All of Us), transparency in method development, and focus on operationalizing a practical rurality scale. I present some opportunities for improvement:

**Response:** Thank you for your thoughtful review and constructive feedback. We have made every effort to address comments as effectively as possible, and you can find our responses below.

**Comment 2:** Provide justification or sensitivity testing for the thresholds used in survey coding.

**Response:** Thank you for your suggestion. We have provided justification for thresholds used in the survey coding and modified the statement (Lines 155 – 158) by adding that “Conservative thresholds were selected through careful expert deliberations to reflect a high burden of barriers in each domain and to capture participant groups with persistent or widespread challenges rather than isolated instances.”

**Comment 3:** Expand discussion comparing this rurality scale with existing measures (e.g., RUCA, IRR, county-level designations).

**Response:** We have expanded the discussion and included a comparison of our rurality scale with existing measures. We emphasized the strength of our scale in addressing compatibility issues common in large-scale de-identified datasets, such as *All of Us*, when compared to existing measures. We assert that “When comparing our rurality scale to existing measures, such as the Rural-Urban Commuting Area (RUCA) codes and the Index of Relative Rurality (IRR), our rurality scale offers a standardized classification system that is compatible with three-digit ZIP codes. It provides a strong starting point for researchers studying rurality in de-identified large-scale datasets such as the *All of Us* Research Program, as it enables triangulation with survey questions, EHR data, and other factors to systematically group participants by rurality. ” (Lines 277 – 282)

**Comment 4:** Consider additional analyses (e.g., logistic regression) to test associations while controlling for confounders.

**Response:** We are considering additional analyses to demonstrate the applicability of the rurality scale for research studies that include rurality-related measures, as well as evaluation efforts such as participation and recruitment from rural areas in biomedical and public health research. These analyses are planned for follow-up dissemination efforts.

**Comment 5**: Clarify limitations related to the use of 3-digit ZIP codes and potential ecological bias.

**Response:** In the “Limitations and future work” section, we clarified that “The limited precision of three-digit ZIP codes may lead to misclassification by masking local heterogeneity and factors such as remoteness and travel distance to health resources.” (Lines 306 – 308)

**Comment 6:** Expand figure captions to allow interpretation without reference to the main text.

**Response:** We have updated the figure captions for further clarity.

**Comment 7:** Proofread for minor grammatical and stylistic improvements.

**Response:** We have proofread the manuscript to address any editorial and grammatical errors and improve the overall organization.

**Response to Reviewer 2**

**Comment 1: Introduction section**

The introduction moves logically from the general problem of rural health to the specific challenge of defining rurality, to the limitations of the All of Us dataset, and finally, to the study's proposed solution. However, while the paper clearly states the purpose, the final sentences of the introduction could be strengthened to more explicitly highlight the broader implications of the work. For example, in addition to informing future research, how might this rurality scale be used by policymakers or public health organizations? Also, the introduction uses several citations to support its claims. You could consider starting the introduction with a single, compelling statistic about the life expectancy gap between rural and urban populations to immediately grab the reader's attention and underscore the urgency of the problem.

**Response:** Thank you for your feedback and suggestions. We have addressed the broader implications of the work and revised the introduction section as suggested.

**Comment 2: Methods**

This is a well-structured and detailed "Methods" section that clearly outlines the study's approach. It effectively explains the different tiers of the All of Us dataset and, most importantly, provides a transparent, step-by-step process for creating the rurality scale. To further strengthen the methods section, there should be a justification for the threshold. **For instance, i**n the section on "Healthcare access and utilization survey data preparation and analysis," the authors state that thresholds of "six or more affirmative responses" for delayed care and "nine or more affirmative responses" for affordability were selected to reflect a "high burden." This is a critical point that needs more robust justification. Authors should explain the rationale behind these specific numbers. Were they based on a pre-existing clinical standard, a statistical analysis (e.g., a percentile cutoff), or a consensus among the research team? Providing a clearer justification will strengthen the validity of the findings.

**Response:** We have clarified the justification for the threshold by providing a more robust explanation, including consensus among the domain experts and the research team.

**Comment 3: There must be consistency in Language:** The term "rurality" is used, but the scale is also referred to as a "rural-urban continuum" in the figure caption. While these are related, using consistent terminology throughout the paper will prevent any potential confusion.

**Response:** We have modified Fig 3. caption to ensure consistency in the use of the “rurality terminology.

### Comment 4: Discussion Section

The discussion correctly re-establishes the context by referencing the existing literature on rurality definitions. It effectively highlights the study's unique methodological strength—the creation of a continuous rurality scale compatible with de-identified data. However, here is a suggestion for revision:

Consider reorganizing the discussion to maximize its impact. A potential flow could be:

1. **Summary of Key Findings:** Start with a concise summary of your most significant findings from the results section. For example, "This study shows that participants living in areas with higher rurality percentages, as defined by our novel scale, report statistically significant disparities in delayed care and healthcare affordability compared to their urban counterparts."
2. **Connection to Literature and Contribution:** Next, explain how these findings align with or contradict existing research. Reiterate your study's unique contribution—the development of a practical rurality scale—in this context.
3. **Limitations and Implications:** Acknowledge any limitations of your methodology (e.g., reliance on 3-digit ZIP codes). Then, discuss the broader implications of your findings for patient care, policy, and research.
4. **Future Work:** Conclude by providing a clear and detailed roadmap for future research using your developed scale.

**Response:** Thank you for the suggestion. We have carefully reorganized the “Discussion” with more depth and coherence based on your suggestion. We also included a “Limitations and future work” section and made it more robust.

**Comment 5: Conclusion**

This is a strong conclusion that effectively summarizes the study's key contributions. It brings the paper full circle by re-emphasizing the problem and presenting the proposed rurality scale as a viable solution.

**Critique of the Conclusions Section**

- **Strengths:** The conclusion is concise and effectively restates the study's main purpose and findings. It correctly identifies the potential of the rurality scale to expand the scope and quality of research within the All of Us program.
- **Areas for Improvement:**
  - **Specificity:** The conclusion mentions "potential and current gaps" and "associations between rurality, delayed care, and healthcare affordability" without directly stating the findings. It would be stronger to explicitly state what was found.
  - **Proposed Revision:** Start with a more direct summary of the results. "In this study, we developed a standardized approach to identify and characterize rural participants within the All of Us program and found that higher rurality was significantly associated with increased reports of delayed care and difficulty with healthcare affordability."
  - **"So What?" Moment:** The last sentence is good, but it can be enhanced to leave a lasting impression. What is the ultimate goal of this work? It's not just about "quantifying representation"—it's about improving health equity.
  - **Proposed Revision:** The final sentence could be more ambitious. This work provides a critical foundation for future investigations, not only into how rurality intersects with social determinants of health but also for developing targeted interventions to improve health outcomes and reduce health disparities in rural America.

**Response:** We have carefully included statements that strengthen the “Conclusions” section based on your suggestion.

**Changes to References**

To address the reviewers’ comments, we incorporated new references into the manuscript, which increased the total citation count from 27 in the previous submission to 31 in the revised version.

**Data Availability**

We appreciate the request to upload our dataset to a public repository. However, the data analyzed in this study are from the NIH *All of Us* Research Program, which is a controlled-access, third-party repository. Under the program’s Data and Statistics Dissemination Policy, registered users are prohibited from exporting or redistributing participant-level data outside of the Researcher Workbench. For this reason, we are unable to deposit the minimal dataset into public repositories. Instead, we have ensured reproducibility by directing interested researchers to obtain the full dataset directly through the *All of Us* Researcher Workbench (https://www.researchallofus.org/data-tools/workbench/), where access can be granted to eligible researchers who complete the registration process. This approach aligns with how prior PLOS publications using *All of Us* data have described availability while adhering to NIH data use requirements. We have revised the data availability statement in the manuscript to include additional details that accurately reflect this update.

**Copyright Clarification for Figure 3**

Thank you for your comment regarding Figure 3. We confirm that Figure 3 was created entirely by the authors and does not contain any copyrighted or proprietary material. The map was generated using publicly available, copyright-free data sources and was edited and formatted by the authors to illustrate the findings of this study. Therefore, it is original content and fully compliant with the Creative Commons Attribution License (CC BY 4.0).
